# The impact of mental health literacy on professional psychological help-seeking attitudes among Chinese college students: the chain mediating role of anxiety and depression

**DOI:** 10.3389/fpsyg.2025.1553716

**Published:** 2025-05-27

**Authors:** Ke Shi, Jinlai Tian

**Affiliations:** School of Educational Science, Beihua University, Jilin, China

**Keywords:** mental health literacy, professional psychological help-seeking attitudes, anxiety, depression, university students

## Abstract

By analyzing cross-sectional data from 2,078 college students, this study aims to explore the impact of mental health literacy on Chinese college students’ professional psychological help-seeking attitudes and to test the chained mediation role of anxiety and depression. Results show that mental health literacy significantly positively predicts professional psychological help-seeking attitudes. Anxiety and depression exhibit partial mediation effects, forming a chained mediation pathway, suggesting mental health literacy indirectly reduces depression by alleviating anxiety, enhancing professional psychological help-seeking attitudes. This study reveals the chained mechanism between anxiety and depression, addressing previous neglect of emotional factors, and highlights the core role of practical dimensions in mental health literacy. It is suggested that universities incorporate mental health literacy training into curricula, strengthen practical skill development, and design interventions for emotion regulation to disrupt the negative cycle of anxiety-depression. This study provides a theoretical basis and targeted strategies for optimizing psychological services for college students.

## 1 Introduction

College students, as the core of the younger generation, are in a crucial stage of transitioning from adolescence to adulthood (Liu et al., 2024). However, psychological health issues among college students are frequent due to the lack of social adaptability, the complexity of interpersonal relationships, and the immense academic and employment pressures (Lun et al., 2018; Casey et al., 2022; Wolitzky-Taylor et al., 2020; Chan and Sun, 2021). In the meta-analysis, the detection rates for various mental health issues among college students in mainland China were as follows: 13.7% for anxiety, 20.8% for depression, 23.5% for sleep problems, 4.5% for somatization, 10.8% for suicidal ideation, 16.2% for self-harm, and 2.7% for suicide attempts (Chen et al., 2022). Psychological health issues not only affect the personal development of college students but may also have long-term negative impacts on their future careers and social adaptation. Therefore, it is particularly important to enhance college students’ awareness and coping abilities regarding mental health issues.

Significant progress and innovation have been made in the current educational approaches to enhancing college students’ awareness of mental health issues. For example, Chookerd et al. (2025) conducted a randomized controlled trial and found that, compared to traditional teaching methods, motion-graphic media significantly improved students’ awareness and understanding of mental health problems. However, college students’ ability to independently cope with mental health issues remains somewhat insufficient, and they often require external support (Patias et al., 2021; Buizza et al., 2022; Grubic et al., 2020). In 2011, the General Office of the Ministry of Education of China issued a notice on the “Basic Standards for Psychological Health Education Work in Colleges and Universities (Trial),” which pointed out that universities should establish psychological counseling centers to provide counseling services to students, in line with industry requirements. Furthermore, in 2017, the Ministry of Education of China clearly mandated in the “Implementation Outline for the Quality Improvement of Ideological and Political Work in Universities” that universities must have at least one full-time psychological health educator for every 4,000 students, with a minimum of two such teachers at each university.

When individuals encounter psychological distress, they often seek help from various sources. These may include professionals, such as psychiatrists and psychological counselors, as well as non-professionals, like family members and friends (Skrobinska et al., 2024; Rickwood and Thomas, 2012). Family and friends can provide comfort and support to those dealing with mental health challenges. In contrast, psychiatrists and psychological counselors offer more specialized and effective solutions for addressing mental health issues. A study indicates that only 8.2% of university students in China diagnosed with psychological disorders have sought professional mental health treatment (Liu et al., 2016). Additionally, a survey found that only about 1% of college students at universities have utilized counseling services at mental health centers (Tao and Yao, 2015). Despite ongoing improvements in mental health services at university counseling centers, such as increasing the number of full-time counselors and streamlining the appointment process for psychological counseling, the uptake remains low (Oswalt et al., 2020). Many college students dealing with mental health issues often seek support from non-professionals instead of turning to professional psychological help. Consequently, they may avoid confronting their psychological challenges or may not seek help at all (Disabato et al., 2018; Pace et al., 2018; Li et al., 2016; Liu et al., 2017).

Therefore, it is necessary to explore the psychological mechanisms underlying college students’ reluctance to seek help and to enhance the utilization of mental health services among Chinese college students. This study, involving 2078 college students in China, aims to examine how mental health literacy influences individuals’ professional psychological help-seeking attitudes through indirect effects on negative emotions using a chained mediation model. Previous studies have primarily focused on the impact of cognitive and behavioral factors on professional psychological help-seeking attitudes, neglecting the potential role of emotional factors (Shi et al., 2020; Aguirre Velasco et al., 2020). This study investigates anxiety and depression as “chained mediators” in the pathway between mental health literacy and professional psychological help-seeking attitudes, and conducts a detailed analysis of the six dimensions of mental health literacy. It provides a new perspective for theoretical research on professional psychological help-seeking attitudes and offers specific and effective intervention ideas for improving such attitudes among college students.

## 2 Literature review and research hypothesis

### 2.1 Mental healthy literacy and professional psychological help-seeking attitudes

Mental health literacy (MHL) refers to an individual’s understanding and attitudes toward mental health, which contribute to identifying, managing, and preventing mental health issues (Jorm et al., 1997; Ma, 2019; Galyautdinova et al., 2022). Mental health literacy levels also vary across groups with differences in gender, age, education, urban–rural status, and culture (Furnham and Swami, 2018). Studies have shown that the mental health literacy of the Chinese population is generally lower than that of other cultural groups, particularly in terms of recognizing certain mental disorders and having a limited understanding of standard psychological treatment methods (Wong et al., 2012; Jiang et al., 2021). Zhang et al. (2024) found that the mental health literacy of first-year students at a university in China was at a moderate level. However, there is potential for improvement, especially among female students, those who have taken mental health education courses, and those who have read books related to psychology, as they showed higher levels of mental health literacy.

Professional psychological help-seeking attitudes (PPHSA) refer to a person’s willingness to consult mental health professionals, such as psychiatrists, psychological counselors, and psychotherapists, when dealing with mental health issues (Wu et al., 2014; Kong and Hao, 2021). Research findings have indicated that factors contributing to individuals’ reluctance to seek professional psychological help encompass low perceived need, skepticism towards the effectiveness of treatment, distrust of psychological professionals, lack of confidence in professional psychological assistance, or an attempt to disregard mental health issues (Chen, 2018; Fukuda et al., 2016; Chen and Kok, 2017; Lumaksono et al., 2020). These factors are all associated with mental health literacy (Aguirre Velasco et al., 2020; Ma, 2019). Jung et al. (2017) conducted a study in the United States and found that an individual’s level of mental health literacy impacts their professional psychological help-seeking attitudes. Specifically, higher mental health literacy is associated with more positive professional psychological help-seeking attitudes. Jia et al. (2023) conducted a study on Chinese college students and found a reassuring correlation. They discovered that during the pandemic, students with higher mental health literacy were more likely to seek professional psychological help. Similarly, Yang et al. (2024) found that mental health literacy significantly and positively predicts the likelihood of Chinese university students engaging in professional psychological help-seeking behaviors. Based on this, the following hypothesis is proposed:

*H1*: Mental health literacy can significantly positively predict professional psychological help-seeking attitudes.

### 2.2 The mediating role of anxiety and depression

The ABC theory of emotions posits that the activating event (A) is an indirect cause of an individual’s emotional and behavioral responses, while the cognition and evaluation (B) resulting from the activating event are the more direct causes of the individual’s emotional and behavioral responses (Criddle, 1977). Therefore, mental health literacy may indirectly influence an individual’s attitude toward professional psychological help-seeking by triggering emotional states (Galyautdinova et al., 2022; Carvalho et al., 2022; Günaydin, 2024; Ramos-Galarza et al., 2024). Tuan et al. (2024) employed machine learning for detecting depression, anxiety, and stress symptoms in individuals recovering from COVID-19. Their study not only demonstrated the potential of technology in monitoring mental health, but also highlighted the prevalence and significance of these emotions during the recovery process. This further supports the role of emotional factors as a bridge between the identification of mental health issues and help-seeking behavior. Anxiety and depression are two typical negative emotions. Anxiety is an unpleasant emotional state marked by inner restlessness and a premonition of potential negative situations that are hard to handle (Dobson, 1985). Depression is a negative emotional state characterized by feelings of deep sadness, loss of interest, and a sense of worthlessness (Beck and Haigh, 2014).

Studies have shown that there is a negative correlation between individual mental health literacy and anxiety or depression, and the correlation is more substantial in young people (Huang et al., 2021; Zhong et al., 2024). Foster et al. (2023) conducted a longitudinal study. They discovered that adolescents experiencing symptoms of anxiety or depression are more likely to adopt negative coping strategies, such as avoidance when faced with stressors. This finding suggests that anxiety and depression may influence an individual’s willingness to seek professional psychological help. Furthermore, it implies that these negative emotional states could mediate the relationship between mental health literacy and professional psychological help-seeking attitudes. Based on this, this paper proposes the following hypothesis:

*H2*: Anxiety and depression each mediate the relationship between mental health literacy and professional psychological help-seeking attitudes.

### 2.3 The chain mediating effect of anxiety and depression

Anxiety and depression exhibit high comorbidity and correlation (De la Torre-Luque et al., 2020; Tao et al., 2024), and past research has often examined both anxiety and depression as negative emotions together (Young et al., 2019; Huang et al., 2021). However, some studies have found that certain types of anxiety can serve as predictors of depression, such as existential anxiety, attachment anxiety, and social anxiety, all of which can significantly positively predict individual depression (Wang and Yu, 2022; Chen and Wang, 2013; Flynn et al., 2019). Yildirim et al. (2021) found that anxiety significantly predicted depression among the elderly population. The unique characteristic of anxiety lies in the overestimation of thoughts concerning threats and dangers, whereas depression is distinctively marked by negative evaluations of oneself, as well as past and future events (Garnefski and Kraaij, 2018). Prolonged exposure to anxiety results in the continuous accumulation of psychological stress within an individual. If this stress remains unmitigated, it may lead to a loss of interest and confidence in various aspects of life, ultimately precipitating a depressive state. This cumulative effect of psychological stress constitutes one of the pivotal factors through which anxiety predicts depression (Lee et al., 2020). Therefore, as typical negative emotions, anxiety, and depression are likely to play different roles in the relationship between mental health literacy and professional psychological help-seeking attitudes. Based on this, this paper proposes the following hypothesis:

*H3*: Anxiety and depression mediate the relationship between mental health literacy and professional psychological help-seeking attitudes in a chain mediation model.

## 3 Methods

### 3.1 Participants

This study utilized the questionnaire star platform to conduct a cross-sectional study of college students from three colleges and universities in Northeast China, using cluster stratified sampling. After obtaining informed consent from the participants, they were invited to complete the questionnaire online. A total of 2,183 college students participated in the study. Following the exclusion of data with inconsistent responses or response times less than 150 s or longer than 1800 s, the final valid sample consisted of 2,078 participants with an average age of 19.29 ± 1.38 years. The effective response rate of the questionnaire was 95.19%. The study was approved by the Ethics Committee of Beihua University (NO: 20240925).

The final statistical analysis sample included 1,414 females (68%) and 664 males (32%); 965 freshmen (46.4%), 641 sophomores (30.8%), 436 juniors (21%), and 36 seniors (1.7%); 497 only children (23.9%) and 1,581 non-only children (76.1%). As shown in Table 1 1,372 university students reported experiencing left-behind situations (17.9%). In comparison, only 128 university students had professional psychological help-seeking experiences, including hospital psychology departments, psychological counseling agencies, and school counseling centers (6.2%), as shown in Table 1.

**Table 1:** Descriptive of socio-demographic.

	Total *N* (%)
Gender	
Male	664 (32.0)
Female	1,414 (68.0)
Grade	
Freshman (1 st year)	965 (46.4)
Sophomore (2nd year)	641 (30.8)
Junior (3rd year)	436 (21.0)
Senior (4th year)	36 (1.7)
Only child	
Yes	497 (23.9)
No	1,581 (76.1)
Left-behind experiences	
Yes	372 (17.9)
No	1,706 (82.1)
Professional psychological help-seeking experiences	
Yes	128 (6.2)
No	1,950 (93.8)

*N* = 2078.

### 3.2 Measures

#### 3.2.1 Mental health literacy

The Chinese version of the Mental Health Literacy Scale (MHLS) was used to measure the mental health literacy of university students (Ma, 2019). This scale was initially developed by O'Connor and Casey (2015) and later adapted into Chinese by Ma (2019). After revision, the scale consists of 35 items across six dimensions: the ability to identify mental disorders (8 items), knowledge of disease risk and causes (2 items), knowledge of self-therapy (2 items), knowledge of how to access professional help (3 items), knowledge of how to gain mental health information (4 items), and attitudes that promote disease awareness or help-seeking behaviors (16 items). The first four dimensions use a 4-point scale, with three items being reverse-scored; the last two dimensions use a 5-point Likert scale, with nine items being reverse-scored. The total score on the scale ranges from 35 to 160, with higher scores indicating higher mental health literacy. Zhang et al. (2024) reported a Cronbach’s *α* coefficient of 0.79 for the scale. In this study, the Cronbach’s α coefficient of the scale was 0.84.

#### 3.2.2 Professional psychological help-seeking attitudes

The Chinese version of the Attitude Toward Seeking Professional Psychological Help Short Form (ATSPPH-SF) was used to measure university students’ professional psychological help-seeking attitudes (Kong and Hao, 2018). Fischer and Turner (1970) originally developed the scale, which was later localized by Chinese scholars Kong and Hao (2018). It consists of 10 items covering three dimensions: openness of help-seeking (3 items), effectiveness of help-seeking (4 items), and independence in help-seeking (3 items). The scale uses a 4-point Likert scale, where participants rate each item from 0 (disagree) to 3 (agree), with five items being reverse-scored. The total score ranges from 0 to 30, with higher scores indicating more positive attitudes toward seeking professional psychological help. Kong and Hao (2021) reported a Cronbach’s *α* coefficient of 0.76 for the scale. In this study, the Cronbach’s α coefficient of the scale was 0.77.

#### 3.2.3 Anxiety and depression

The Anxiety and Depression subscales from the Depression Anxiety and Stress Scale (DASS) were used to measure the anxiety and depression levels of university students (Gong et al., 2010). Lovibond (1995) initially developed the scale, which was later localized by Gong et al. (2010). The depression and anxiety subscales each consist of 7 items. They use a 4-point Likert scale, with participants rating each item from 0 (not applicable) to 3 (always applicable). Higher scores on each subscale indicate higher levels of depression or anxiety in the individual. Han et al. (2022) reported a Cronbach’s α coefficient of 0.82 for the anxiety subscale and 0.87 for the depression subscale. In this study, Cronbach’s α coefficient for the anxiety subscale was 0.86, and for the depression subscale, it was 0.88.

### 3.3 Statistical analyses

The analysis was conducted using SPSS 27.0 for reliability and validity assessment, standard method bias testing, descriptive statistics, and correlation analysis. Mediation analysis was performed using Model 6 from the SPSS macro program PROCESS 3.3 plugin (Hayes, 2017).

## 4 Result

### 4.1 Common method bias test

The Harman single-factor test results showed that there were 11 factors with eigenvalues greater than 1. The variance explained by the first factor was 17.44%, which is below the standard threshold of 40%, indicating that the common method bias test was passed (Zhou and Long, 2004). Therefore, this study does not have a significant standard method bias issue.

### 4.2 Descriptive statistics and correlational analysis

This study conducted a differential test on demographic variables and found significant differences in college students’ professional psychological help-seeking attitudes based on gender (*t* = −4.141, *p* < 0.001), age (*F* = 2.206, *p* < 0.05), whether they have had left-behind experiences (*t* = −4.571, *p* < 0.001), and whether they have had professional psychological help-seeking experiences (*t* = −4.734, *p* < 0.001), as shown in Table 2. Therefore, these demographic variables need to be controlled as covariates in the subsequent statistical analysis of this study.

**Table 2:** Differential testing.

Variable	*t*	df	*F*	*p*-value
Gender	−4.141^***^			<0.001
Age		12	2.206^**^	0.010
Grade		3	2.063	0.103
Only Child	1.823			0.069
Left-behind experiences	−4.571^***^			<0.001
Professional psychological help-seeking experiences	−5.083^***^			<0.001

*N* = 2078, ***p < 0.001, **p < 0.01.

Pearson’s correlation analysis results show that significant correlations exist between mental health literacy, anxiety, depression, and professional psychological help-seeking attitudes. Mental health literacy has a significant positive correlation with professional psychological help-seeking attitudes (*r* = 0.428, *p* < 0.001). Mental health literacy shows significant negative correlations with both anxiety and depression (*r* = −0.136, *p* < 0.001; *r* = −0.165, *p* < 0.001). Professional psychological help-seeking attitudes show significant negative correlations with both anxiety and depression (*r* = −0.415, *p* < 0.001; *r* = −0.421, *p* < 0.001). Anxiety and depression have a significant positive correlation (*r* = 0.818, *p* < 0.001), as shown in Table 3.

**Table 3:** Descriptive statistics and correlational analysis.

Variable	*M*	*SD*	1	2	3	4	5	6	7	8	9	10	11	12	13
1. Anxiety	3.06	3.299	1												
2. Depression	2.69	3.312	0.818^***^	1											
3. Professional psychological help-seeking attitudes	18.18	4.100	−0.415^***^	−0.421^***^	1										
4. Openness to help-seeking	5.90	1.640	−0.445^***^	−0.447^***^	0.827^***^	1									
5. Effectiveness of help-seeking	7.53	1.950	−0.299^***^	−0.309^***^	0.758^***^	0.465^***^	1								
6. Independence in help-seeking	4.75	1.742	−0.223^***^	−0.224^***^	0.727^***^	0.485^***^	0.226^***^	1							
7. Mental health literacy	98.64	10.959	−0.136^***^	−0.165^***^	0.428^***^	0.377^***^	0.362^***^	0.247^***^	1						
8. The ability to identify mental disorders	24.64	3.704	0.016	−0.008	0.206^***^	0.148^***^	0.272^***^	0.040	0.580^***^	1					
9. Knowledge of disease risk and causes	4.28	0.792	0.054^*^	0.044^*^	0.008	0.007	0.002	0.010	0.186^**^	0.143^***^	1				
10. Knowledge of self-therapy	4.14	0.577	−0.059^**^	−0.099^***^	0.103^***^	0.101^***^	0.075^**^	0.064^**^	0.154^***^	0.055^*^	0.061^**^	1			
11. Knowledge of how to access to professional help	7.20	0.939	−0.020	−0.037	0.161^***^	0.143^***^	0.172^***^	0.051^*^	0.379^***^	0.341^***^	0.155^***^	−0.003	1		
12. Knowledge of how to gain mental health information	14.91	2.692	−0.219^***^	−0.215^***^	0.341^***^	0.298^***^	0.384^***^	0.091^***^	0.539^***^	0.355^***^	0.067^**^	0.078^***^	0.288^***^	1	
13. Attitudes that promote disease awareness or help-seeking behaviors	43.47	7.990	−0.118^***^	−0.143^***^	0.350^***^	0.323^***^	0.215^***^	0.278^***^	0.847^***^	0.154^***^	0.044^*^	0.081^***^	0.133^***^	0.192^***^	1

*N* = 2078, ^*^p < 0.05, ^**^p < 0.01, ^***^p < 0.001. Mental health literacy includes six dimensions: the ability to identify mental illness, knowledge of disease risks and causes, knowledge of self-therapy, knowledge of how to access to professional help, knowledge of how to gain mental health information and attitudes that promote disease awareness or help-seeking behaviors. Professional psychological help-seeking attitudes include three dimensions: openness to help-seeking, effectiveness of help-seeking and independence in help-seeking.

In addition, there are significant correlations between the three dimensions of mental health literacy, anxiety, depression, and professional psychological help-seeking attitudes. Knowledge of self-therapy is significantly positively correlated with professional psychological help-seeking attitudes (*r* = 0.103, *p* < 0.001). Knowledge of self-therapy is significantly negatively correlated with both anxiety and depression (*r* = −0.059, *p* < 0.001; *r* = −0.099, *p* < 0.001). Knowledge of how to gain mental health information is significantly positively correlated with professional psychological help-seeking attitudes (*r* = 0.341, *p* < 0.001). Knowledge of how to gain mental health information is significantly negatively correlated with both anxiety and depression (*r* = −0.219, *p* < 0.001; *r* = −0.215, *p* < 0.001). Attitudes that promote disease awareness or help-seeking behaviors are significantly positively correlated with professional psychological help-seeking attitudes (*r* = 0.350, *p* < 0.001). Attitudes that promote disease recognition or help-seeking behaviors are significantly negatively correlated with both anxiety and depression (*r* = −0.118, *p* < 0.001; *r* = −0.143, *p* < 0.001). However, the ability to identify mental disorders and knowledge of how to access professional help does not show significant correlations with anxiety or depression. Additionally, knowledge of disease risks and causes does not show a significant correlation with professional psychological help-seeking attitudes, as shown in Table 3.

### 4.3 Mediation analyses

**Figure 1:**
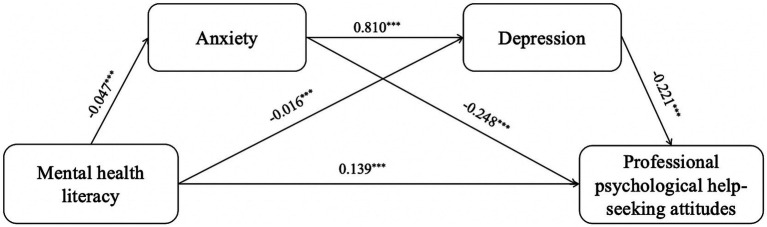
The serial mediation of anxiety and depression. ^*^p < 0.05, ^**^p < 0.01, ^***^p < 0.001. Controlling gender, age, left-behind experiences, professional psychological help-seeking experiences.

After controlling for gender, age, experience of being left behind, and professional psychological help-seeking experience, the mediation analysis results indicate that Mental health literacy significantly positively predicts professional psychological help-seeking attitudes (*β* = 0.139, *p* < 0.001). Anxiety significantly negatively predicts professional psychological help-seeking attitudes (*β* = −0.248, *p* < 0.001). Depression significantly negatively predicts professional psychological help-seeking attitudes (*β* = −0.221, *p* < 0.001), as shown in Table 4. Furthermore, the results of this study show that the 95% CI for the direct effect of mental health literacy on professional psychological help-seeking attitudes [0.018, 0.030] does not include 0, indicating a significant direct effect. The 95% CI for the indirect path 1 (mental health literacy → anxiety → professional psychological help-seeking attitudes) [0.007, 0.017] does not include 0, indicating a significant indirect effect 1, with a mediation effect accounting for 7.362%. The 95% CI for the indirect path 2 (mental health literacy → depression → professional psychological help-seeking attitudes) [0.002, 0.006] does not include 0, indicating a significant indirect effect 2, with a mediation effect accounting for 2.454%. The 95% CI for the indirect path 3 (mental health literacy → anxiety → depression → professional psychological help-seeking attitudes) [0.005, 0.013] does not include 0, indicating a significant indirect effect 3, with a chain mediation effect accounting for 4.908%. Therefore, the mediation effects of anxiety and depression, as well as their chain mediation effect, are valid between mental health literacy and professional psychological help-seeking attitudes, as shown in Table 4 and Figure 1.

**Table 4:** Testing the mediation effect of mental health literacy on professional psychological help-seeking attitudes.

**Predictors**	**Anxiety**		**Depression**		**Professional psychological help-seeking attitudes**		
	***β***	***t***	***β***	***t***	***β***	***t***	
Mental health literacy	−0.047	−7.428^***^	−0.016	−4.109^***^	0.139	20.080^***^	
Anxiety			0.810	60.784^***^	−0.248	−6.266^***^	
Depression					−0.221	−5.662^***^	
*R* ^2^	0.111		0.675		0.330		
*F*	51.617^***^		717.289^***^		145.528^***^		
Knowledge of self-therapy	−0.310	−2.584^**^	−0.292	−4.048^***^	0.475	3.387^***^	
Anxiety			0.816	61.921^***^	−0.273	−6.319^***^	
Depression					−0.279	−6.552^***^	
*R* ^2^	0.090		0.675		0.204		
*F*	40.993^***^		717.034^***^		75.640^***^		
Knowledge of how to gain mental health information	−0.270	−10.755^***^	−0.045	−2.828^**^	0.387	13.051^***^	
Anxiety			0.810	59.810^***^	−0.218	−5.231^***^	
Depression					−0.259	−6.313^***^	
*R* ^2^	0.135		0.674		0.260		
*F*	64.871^***^		712.767^***^		103.980^***^		
Attitudes that promote disease awareness or help-seeking behaviors	−0.054	−6.270^***^	−0.019	−3.547^**^	0.152	15.584^***^	
Anxiety			0.813	61.129^***^	−0.255	−6.234^***^	
Depression					−0.243	−6.018^***^	
*R* ^2^	0.104		0.675		0.283		
*F*	48.136^***^		715.098^***^		116.924^***^		

*N* = 2078, ^***^p < 0.01. Controlling gender, age, left-behind experiences, professional psychological help-seeking experiences.

**Figure 2:**
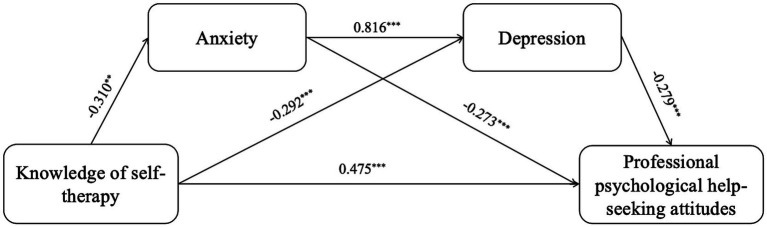
The serial mediation of anxiety and depression. ^*^p < 0.05, ^**^p < 0.01, ^***^p < 0.001. Controlling gender, age, left-behind experiences, professional psychological help-seeking experiences.

Across the various dimensions of mental health literacy, the 95% CI for the direct effect of knowledge of self-therapy on professional psychological help-seeking attitudes [0.200, 0.751] does not include 0, indicating a significant direct effect. The 95% CI for the indirect path 1 (knowledge of self-therapy → anxiety → professional psychological help-seeking attitudes) [0.016, 0.164] does not include 0, indicating a significant indirect effect 1, with a mediation effect accounting for 11.921%. The 95% CI for the indirect path 2 (knowledge of self-therapy → depression → professional psychological help-seeking attitudes) [0.033, 0.140] does not include 0, indicating a significant indirect effect 2, with a mediation effect accounting for 11.501%. The 95% CI for the indirect path 3 (knowledge of self-therapy → anxiety → depression → professional psychological help-seeking attitudes) [0.013, 0.141] does not include 0, indicating a significant indirect effect 3, with a chain mediation effect accounting for 9.958%. Therefore, the mediation effects of anxiety and depression, as well as their chain mediation effect, are valid between knowledge of self-therapy and professional psychological help-seeking attitudes, as shown in Table 5 and Figure 2.

**Table 5:** Direct and indirect effects.

Outcome	*β*	BootSE	BootLLCI	BootULCI
Direct effect				
Mental health literacy—Professional psychological help-seeking attitudes	0.139	0.007	0.018	0.030
Indirect effect				
Mental health literacy—Anxiety—Professional psychological help-seeking attitudes	0.012	0.002	0.007	0.017
Mental health literacy—Depression—Professional psychological help-seeking attitudes	0.004	0.001	0.002	0.006
Mental health literacy—Anxiety—Depression—Professional psychological help-seeking attitudes	0.008	0.002	0.005	0.013
Direct effect				
Knowledge of self-therapy—Professional psychological help-seeking attitudes	0.475	0.140	0.200	0.751
Indirect effect				
Knowledge of self-therapy—Anxiety—Professional psychological help-seeking attitudes	0.085	0.038	0.016	0.164
Knowledge of self-therapy—Depression—Professional psychological help-seeking attitudes	0.082	0.028	0.033	0.140
Knowledge of self-therapy—Anxiety—Depression—Professional psychological help-seeking attitudes	0.071	0.032	0.013	0.141
Direct effect				
Knowledge of how to gain mental health information—Professional psychological help-seeking attitudes	0.387	0.030	0.329	0.446
Indirect effect				
Knowledge of how to gain mental health information—Anxiety—Professional psychological help-seeking attitudes	0.059	0.013	0.035	0.085
Knowledge of how to gain mental health information—Depression—Professional psychological help-seeking attitudes	0.012	0.005	0.003	0.022
Knowledge of how to gain mental health information—Depression—Professional psychological help-seeking attitudes	0.057	0.011	0.036	0.080
Direct effect				
Attitudes that promote disease awareness or help-seeking behaviors—Professional psychological help-seeking attitudes	0.152	0.010	0.133	0.171
Indirect effect				
Attitudes that promote disease awareness or help-seeking behaviors—Anxiety—Professional psychological help-seeking attitudes	0.014	0.003	0.008	0.020
Attitudes that promote disease awareness or help-seeking behaviors—Depression—Professional psychological help-seeking attitudes	0.005	0.002	0.002	0.008
Attitudes that promote disease awareness or help-seeking behaviors—Anxiety—Depression—Professional psychological help-seeking attitudes	0.011	0.003	0.006	0.016

*N* = 2078, Controlling gender, age, left-behind experiences, professional psychological help-seeking experiences.

**Figure 3:**
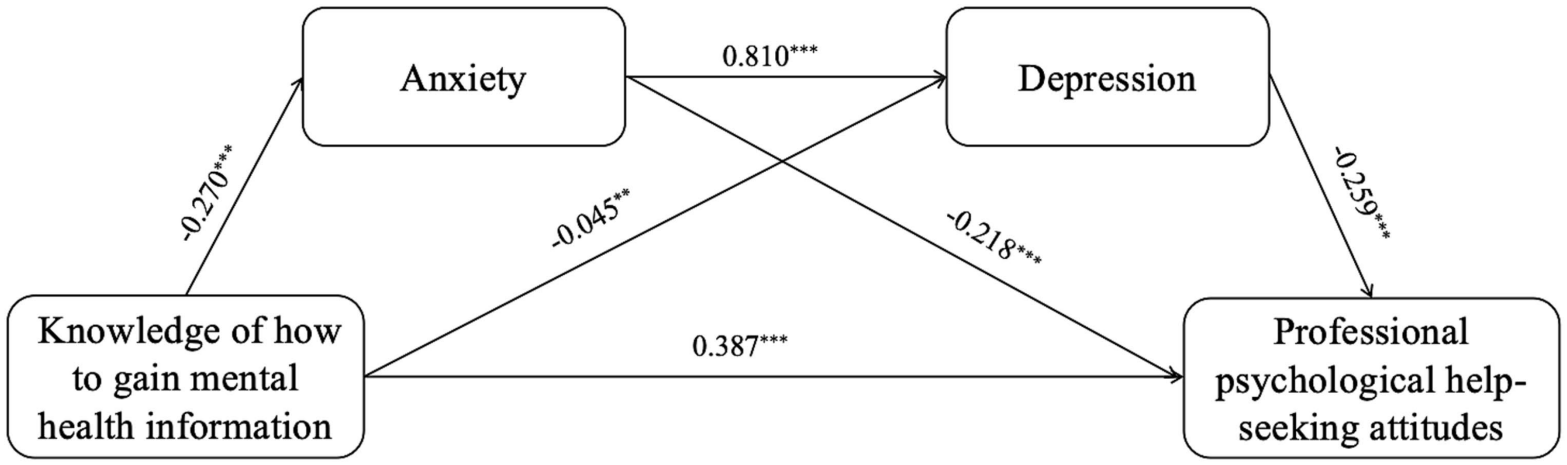
The serial mediation of anxiety and depression. ^*^p < 0.05, ^**^p < 0.01, ^***^p < 0.001. Controlling gender, age, left-behind experiences, professional psychological help-seeking experiences.

The 95% CI for the direct effect of knowledge of how to gain mental health information on professional psychological help-seeking attitudes [0.326, 0.446] does not include 0, indicating a significant direct effect. The 95% CI for the indirect path 1 (knowledge of how to gain mental health information → anxiety → professional psychological help-seeking attitudes) [0.035, 0.085] does not include 0, indicating a significant indirect effect 1, with a mediation effect accounting for 11.456%. The 95% CI for the indirect path 2 (knowledge of how to gain mental health information → depression → professional psychological help-seeking attitudes) [0.003, 0.022] does not include 0, indicating a significant indirect effect 2, with a mediation effect accounting for 2.330%. The 95% CI for the indirect path 3 (knowledge of how to gain mental health information → anxiety → depression → professional psychological help-seeking attitudes) [0.036, 0.080] does not include 0, indicating a significant indirect effect 3, with a chain mediation effect accounting for 11.068%. Therefore, the mediation effects of anxiety and depression, as well as their chain mediation effect, are valid between knowledge about knowledge of how to gain mental health information and professional psychological help-seeking attitudes, as shown in Table 5 and Figure 3.

**Figure 4:**
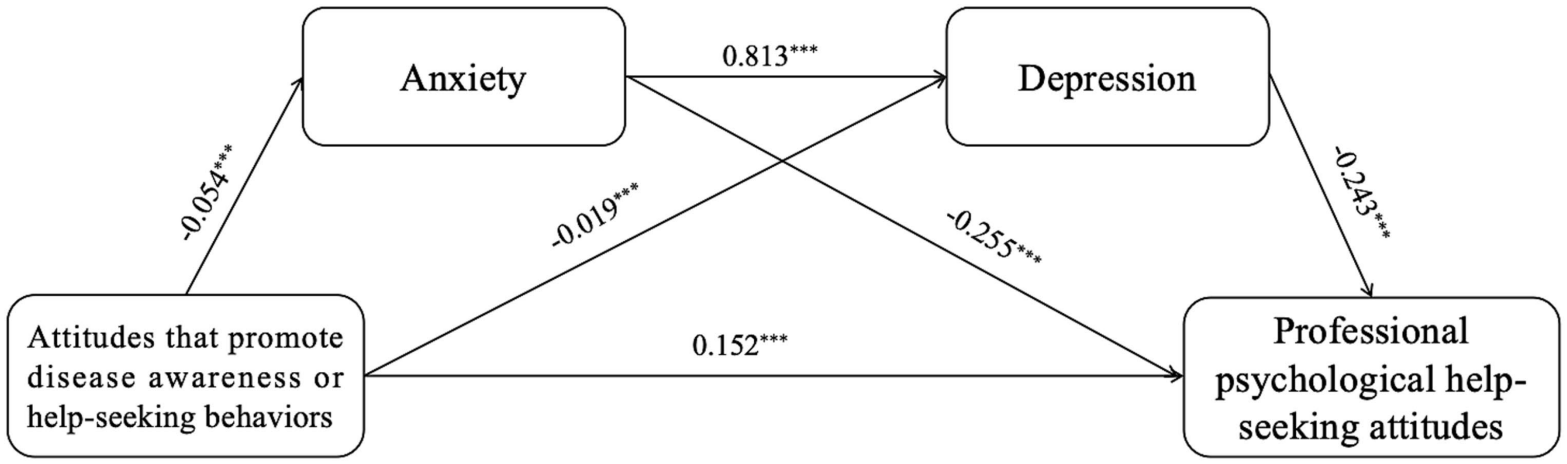
The serial mediation of anxiety and depression. ^*^p < 0.05, ^**^p < 0.01, ^***^p < 0.001. Controlling gender, age, left-behind experiences, professional psychological help-seeking experiences.

The 95% CI for the direct effect of attitudes that promote disease awareness or help-seeking behaviors on professional psychological help-seeking attitudes [0.133, 0.171] does not include 0, indicating a significant direct effect. The 95% CI for the indirect path 1 (attitudes that promote disease awareness or help-seeking behaviors → anxiety →professional psychological help-seeking attitudes) [0.008, 0.020] does not include 0, indicating a significant indirect effect 1, with a mediation effect accounting for 7.692%. The 95% CI for the indirect path 2 (attitudes that promote disease awareness or help-seeking behaviors → depression → professional psychological help-seeking attitudes) [0.002, 0.008] does not include 0, indicating a significant indirect effect 2, with a mediation effect accounting for 2.747%. The 95% CI for the indirect path 3 (attitudes that promote disease awareness or help-seeking behaviors → anxiety → depression → professional psychological help-seeking attitudes) [0.006, 0.016] does not include 0, indicating a significant indirect effect 3, with a chain mediation effect accounting for 6.044%. Therefore, the mediation effects of anxiety and depression, as well as their chain mediation effect, are valid between attitudes that promote disease awareness or help-seeking behaviors and professional psychological help-seeking attitudes, as shown in Table 5 and Figure 4.

## 5 Discussion

### 5.1 Main findings of the present study

The results of this study indicate that the total score of mental health literacy is positively correlated with professional psychological help-seeking attitudes. The five dimensions, “the ability to recognize mental health disorders,” “knowledge of how to access professional help,” “knowledge of self-therapy,” “knowledge of how to gain mental health information,” and “attitudes that promote disease awareness or help-seeking behaviors,” are all positively correlated with professional psychological help-seeking attitudes. However, “knowledge of disease risk and causes” is not correlated with college students’ professional psychological help-seeking attitudes. Among these, the total score of mental health literacy, as well as the three dimensions of “knowledge of self-therapy,” “knowledge of how to gain mental health information,” and “attitudes that promote disease awareness or help-seeking behaviors,” can significantly predict college students’ professional psychological help-seeking attitudes, which confirms hypothesis 1 of this study. Moreover, the results of the mediation analysis indicate that anxiety and depression play both partial mediation and chained mediation roles in the relationship between the total score of mental health literacy and professional psychological help-seeking attitudes, specifically in three dimensions: “knowledge of self-therapy,” “knowledge of how to gain mental health information,” and “attitudes that promote disease awareness or help-seeking behaviors.” These findings validate hypotheses 2 and 3 of the present study.

### 5.2 Comparison with other studies

Mental health literacy is a key predictor of individuals’ professional psychological help-seeking attitudes, which aligns with previous research findings (Yang et al., 2023; Al-Shannaq and Aldalaykeh, 2023; Jia et al., 2023). Individuals with higher levels of mental health literacy are more adept at recognizing symptoms, evaluating treatment efficacy, and overcoming stigma, thereby exhibiting a greater inclination to seek professional psychological help (Gulliver et al., 2010; Rafal et al., 2018). Furthermore, “knowledge of disease risk and causes” did not significantly influence professional psychological help-seeking attitudes, a result that concurs with Fung et al. (2021), who argue that actionable knowledge (e.g., how to access useful resources) is more likely to drive behavioral change than purely theoretical understanding. Lastly, a notable contribution of this study lies in uncovering the chained mediation effect of anxiety and depression between mental health literacy and attitudes towards seeking professional psychological help.

### 5.3 Implication and explanation of findings

#### 5.3.1 The relationship between mental health literacy and professional psychological help-seeking attitudes

Individuals with higher mental health literacy possess knowledge of self-therapy and the ability to identify mental health disorders and generally have a higher perceived need for professional psychological help. They are able to clearly recognize the severity of their mental health issues and assess whether professional treatment is needed (Booth et al., 2017; Salzer, 2022). When faced with mental health issues that require professional help to resolve, they tend to actively seek psychological counseling, which helps to form a positive professional psychological help-seeking attitude (Gulliver et al., 2010; Han et al., 2018; Rafal et al., 2018; Calear et al., 2021). Individuals with lower mental health literacy often struggle to find reliable mental health information or access professional help. As a result, they may lack a clear understanding of mental health issues and treatment options. This gap in knowledge can lead to biases or discrimination against those with mental health disorders (Clough et al., 2019; Fang et al., 2021), which ultimately fosters a negative attitude toward seeking professional psychological help. Moreover, individuals with lower mental health literacy tend to have more negative attitudes that promote disease awareness or help-seeking behaviors. These negative attitudes often stem from mistrust of psychological counselors or psychiatrists, as well as the stigmatization of mental health disorders in society. These factors collectively influence their opposing views on professional psychological help (Chen, 2018; Andrade et al., 2014).

It is worth noting that “knowledge of disease risk and causes” does not show a significant correlation with college students’ professional psychological help-seeking attitudes. Compared to “knowledge of how to access professional help,” “knowledge of self-therapy,” and “knowledge of how to gain mental health information,” “knowledge of disease risk and causes” mainly stays at the cognitive level and lacks sufficient emotional motivation and behavioral guidance. In contrast, “knowledge of how to access professional help,” “knowledge of self-therapy,” and “knowledge of how to gain mental health information” are more practical and actionable, making them more likely to promote positive professional psychological help-seeking attitudes among college students. Therefore, the singular “knowledge of disease risk and causes” may not be an important factor influencing college students’ professional psychological help-seeking attitudes.

#### 5.3.2 The mediation effect of anxiety and depression

First, individuals with higher mental health literacy usually possess more knowledge of self-therapy and how to gain mental health information, which helps them acquire more mental health knowledge and resources. When facing difficulties, they are often able to adapt and thus experience less anxiety and depression quickly (Nguyen Thai and Nguyen, 2018; Cullen et al., 2020). Self-regulation theory refers to the psychological process in which individuals control their cognition, emotions, and behaviors to achieve and maintain an ideal state (Latham and Locke, 1991). When individuals lack mental health knowledge and resources, self-regulation may fail, leading to anxiety or depression (Crisanti et al., 2016; Ding et al., 2022; Mo et al., 2023; Zhong et al., 2024). Similarly, individuals with higher mental health literacy tend to have more positive attitudes that promote disease awareness or help-seeking behaviors. When they experience psychological discomfort, they are more likely to seek external help actively, thereby avoiding the onset of anxiety and depression (Ratnayake and Hyde, 2019). However, while “the ability to identify mental health disorders” and “knowledge of how to access professional help” contribute to the prevention and intervention of mental health issues, they may not be sufficient to predict the occurrence or severity of anxiety and depression directly.

Secondly, from the perspective of attentional bias, individuals with anxiety or depression exhibit attentional bias (Klein et al., 2018), allocating more attentional resources to negative stimuli rather than neutral or positive stimuli (Cisler and Koster, 2010; Sylvester et al., 2016). From a cognitive perspective, individuals with anxiety or depression tend to make more negative evaluations of uncertain events and worry more about the negative consequences such events may bring (Sherman and Ehrenreich-May, 2018; Blanchette and Richards, 2010). Therefore, due to relatively lower self-efficacy in obtaining professional psychological help, individuals with high levels of anxiety or depression tend to focus excessively on barriers to seeking professional psychological help, such as stigma, which may lead them to exhibit more negative professional psychological help-seeking attitudes (Chin et al., 2015; Brenner et al., 2020; Nauphal et al., 2023).

Typically, individuals experience anxiety first when confronted with difficulties rather than depression (Van Kleef and Lelieveld, 2022). Anxiety is more like an alarm signal for psychological discomfort. If negative emotions are not regulated in a timely manner, individuals may fall into a negative emotional cycle, leading to the onset of depression (Wang et al., 2021). Therefore, individuals with low mental health literacy, due to a lack of mental health knowledge and resources, are more likely to experience anxiety, which can then lead to depression. Under the influence of these negative emotions, they may ultimately exhibit more negative professional psychological help-seeking attitudes.

## 6 Strengths, limitations and future direction

Strengths: Firstly, previous studies have predominantly employed small samples to explore the relationship between mental health literacy and professional psychological help-seeking attitudes. In contrast, the present study utilizes a large sample to conduct an in-depth analysis of both direct and indirect influence pathways among the variables, rendering the research findings more generalizable and widely applicable. Secondly, this study identifies dimensions with significant effects (such as “knowledge of self-therapy”), providing more specific and effective insights for interventions aimed at improving college students’ professional psychological help-seeking attitudes. Lastly, the study clarifies the chained mediation effect of anxiety and depression in the relationship between mental health literacy and professional psychological help-seeking attitudes, offering a new theoretical perspective and empirical support for the study of professional psychological help-seeking attitudes.

Limitations and future direction: First, this study is a cross-sectional study, which can only reveal the correlations between variables but cannot explore their causal relationships in depth. Therefore, future research could focus on longitudinal or experimental studies to explore the causal relationships between variables. Second, only a tiny proportion of the university students in this study reported having professional psychological help-seeking experience. Future research could focus on selecting groups with or without professional psychological help-seeking experience to explore the impact of mental health literacy on their professional psychological help-seeking attitudes. Third, anxiety and depression in this study do not refer to specific types, such as attachment anxiety or social depression. Future research could focus on more specific variables to explore how emotions affect professional psychological help-seeking attitudes. Fourth, the indirect pathways between mental health literacy and professional psychological help-seeking attitudes may involve other factors, such as suicidal tendencies and other behavioral factors. This study only examined the mediating roles of anxiety and depression, and future research should broaden the scope of the investigation and explore the relationship between mental health literacy and professional psychological help-seeking attitudes in greater depth, providing empirical support for more effective intervention research. Lastly, the mental health literacy scale used in this study includes six dimensions, but only the chain mediation effects of the three dimensions were found to be valid. This finding suggests that these three dimensions may play a more important role in mental health literacy and also indicates that the measurement tool may have some limitations. Future research can further explore measurement tools for mental health literacy to develop scales more suitable for different populations.

## 7 Conclusion and recommendation

The findings of this study reveal that the overall score of mental health literacy, as well as three specific dimensions - “knowledge of self-therapy,” “knowledge of how to gain mental health information,” and “attitudes that promote disease awareness or help-seeking behaviors” - positively predict professional psychological help-seeking attitudes. Both anxiety and depression serve as partial mediators in the relationship between mental health literacy and professional psychological help-seeking attitudes. Additionally, anxiety and depression exhibit a chained mediation effect in this relationship.

To optimize mental health services for Chinese students, we recommend the following: (a) Incorporate mental health literacy training into university curricula, with an emphasis on developing practical skills (such as how to access useful resources) rather than purely theoretical knowledge. (b) Develop emotion regulation programs aimed at disrupting the chain reactions of anxiety and depression. (c) Reduce the stigma associated with mental health issues through promotional campaigns and promote seeking professional help as a normalized behavior.

## Data Availability

The original contributions presented in the study are included in the article/supplementary material, further inquiries can be directed to the corresponding author/s.
